# The effects of flight training on flying cadets’ brain structure

**DOI:** 10.1371/journal.pone.0313148

**Published:** 2025-02-10

**Authors:** Liang Wang, Chengshi Yang, Dongfeng Yan, Lu Ye, Xi Chen, Shan Ma

**Affiliations:** Institute of Flight Technology, Civil Aviation Flight University of China, Guanghan, Sichuan, China; Universiti Pertahanan Nasional Malaysia, MALAYSIA

## Abstract

In recent years, the impact of professional training on brain structure has sparked extensive research interest. Research into pilots as a high-demand, high-load, and high-cost occupation holds significant academic and economic value. The aim of this study is to investigate the effects of flight training on the brain structure and cognitive functions of flying cadets. The structural magnetic resonance imaging (sMRI) data from 39 flying cadets and 37 general college students underwent analysis using voxel-based morphometry (VBM) and surface-based morphometry (SBM) methods to quantitatively detect and compute multiple indicators, including gray matter volume (GMV), curvature, mean curvature of the white matter surface (MC-WMS), the percentage of surface white matter gray matter (WM-GM percentage), surface Jacobi (S-Jacobi), and Gaussian curvature of white matter surface (GC-WMS). At the voxel level, the GMV in the left temporal pole: middle temporal gyrus region of flying cadets significantly decreased (Gaussian random field, GRF, P < 0.05). At the surface level, there was a significant increase in curvature, MC-WMS, and S-Jacobi in the lateral occipital region of flight cadets (Monte Carlo block level correction, MCBLC, P<0.05), a significant increase in WM-GM percentage in the cuneus region of flight cadets (MCBLC, P<0.05), and a significant increase in GC-WMS in the middle temporal region of flight cadets (MCBLC, P<0.05). In addition, these changes were correlated with behavioral tests. Research suggested that flight training might induce changes in certain brain regions of flying cadets, enabling them to adapt to evolving training content and environments, thereby enhancing their problem-solving and flight abilities. By analyzing multiple indicators at the voxel and surface levels in an integrated manner, it advances our understanding of brain structure, function, and plasticity, while also facilitating a more profound exploration of the neural mechanisms within the pilot’s brain.

## Introduction

Civil aviation transportation assumes a pivotal role in the contemporary transportation system, delivering swift and comfortable services for both international and domestic long-distance travel through a high-speed and expansive route network. It has consequently become an indispensable aspect of modern life. With the rapid advancement of the civil aviation industry, the issue of aviation safety has garnered increasingly significant attention. Among the myriad factors that can jeopardize aviation safety, the human factor stands out due to its broad spectrum and variability. Pilot error, notably, has surpassed equipment failure as the primary cause of civil aviation accidents [1]. Due to the intricate and variable nature of the flight environment, pilots must execute complex flight missions amidst high workload and challenging conditions [2]. These responsibilities demand that pilots possess not only exceptional physical fitness but also outstanding mental and cognitive abilities to manage diverse emergencies and guarantee the safety and efficiency of every flight.

Pilots undergo rigorous selection and training procedures [3]. This process involves thorough and intensive flight training, along with regular assessments, to ensure that each pilot achieves the standards of technical proficiency and specialized knowledge. Flight operations entail a multifaceted process encompassing various phases, such as ground preparation, take-off, climb, cruise, descent, landing, and ground taxiing. During these phases, pilots must sustain heightened concentration, exact mastery of instrument flight techniques, and swift thinking and reactive capabilities to guarantee the secure accomplishment of the flight mission. In summary, aviation serves as both an evaluation of a pilot’s technical aptitude and a trial of their broader qualities and problem-solving abilities. Research indicates that pilots must possess a range of skills, with problem-solving abilities being crucial for maintaining flight safety in hazardous environments [4]. In high-pressure and uncertain environments, swift and precise decision-making and maneuvering are essential to effectively address potential flight challenges. Therefore, problem-solving abilities are essential for pilots and represent a fundamental safeguard to ensure flight safety. The brain serves as the central processor for the pilot’s decision-making and operational functions during the flight mission, underscoring its undeniable importance. It is tasked not only with analyzing intricate flight data and instrument information but also with promptly responding to unforeseen situations and swiftly managing them. Therefore, outstanding pilots must possess not only exceptional technical proficiency but also a brain capable of operating steadily in high-pressure environments. Consequently, neuroimaging techniques, particularly magnetic resonance imaging (MRI), have been extensively utilized in studying the human brain to investigate its operational mechanisms. Consequently, neuroimaging techniques, particularly magnetic resonance imaging (MRI), have been extensively utilized in studying the human brain to investigate its working mechanisms.

In fMRI and diffusion tensor imaging studies, Chen, Xi et al. discovered that flight training correlated with functional connectivity in the brain’s white matter fiber tracts [5], degree centrality [6], and default mode network [7]. These findings suggest that flight training not only enhances the functional connectivity of the brain’s default mode network but also potentially improves cognitive abilities and the decision-making process. Xu, K et al. [8] uncovered that certain brain regions of pilots exhibited increased sensitivity in spontaneous activity within the slow-5 frequency band, offering novel avenues for studying the brain mechanisms of pilots. In addition, Jiang, H et al. [9] observed that the emotional conflict control mechanism of pilots differs from that of ordinary individuals. This distinction may enable pilots to effectively manage emotions and cope with stress more adeptly in high-pressure situations. In terms of sMRI, Adamson, M. M et al. [10] explored the relationship between hippocampal volume and flight training. The findings from this study suggest that flight training could potentially exert a notable influence on brain regions closely associated with learning and memory. Cao, Y et al. [11] detected that the pilot’s hemispheric cortical structure was lateralized, that is, the cortical thickness was asymmetrical. This finding suggests the process of specialization and optimization of the pilot’s brain for performing flight missions. Finally, Xu, K et al. [12] also found that flight training may result in an enlargement of certain brain regions in pilots, potentially enhancing their ability to observe and interpret cockpit instrumentation.

In summary, prior research indicates that flight training may influence the structure and function of the brain, thereby enhancing pilots’ ability to proficiently execute flight missions in high-pressure environments. However, these studies are constrained by several limitations in sample selection, predominantly favoring experienced pilots, which might potentially skew the accuracy of the results. Furthermore, the application of MRI technology in pilot studies remains relatively constrained, primarily concentrating on fMRI and lacking comprehensive exploration of brain structure. Additionally, numerous current studies have depended on a single indicator for analysis, neglecting comprehensive evaluation across multiple indicators. This approach may hinder our understanding of both structural and functional changes in the brain within this context.

Therefore, this paper focuses on flying cadets who have completed their primary training in comparison to regular college students. The primary distinction between the two groups of students lies in the fact that flying cadets undergo professional training encompassing both theoretical courses and practical flight training, which imbues them with unique proficiency in flight skills and flight safety. In contrast, regular college students participate solely in standard theoretical courses and lack practical flight experience. This difference significantly influences variations in cognitive abilities, stress management skills, and career development trajectories between the two groups. We hypothesized that flight training can induce structural changes in certain brain regions of flying cadets, and that these changes may have important effects on their cognitive abilities and flight skills. sMRI is employed to compare the brain structure between the two groups, aiming to investigate the impact of flight training on the brain and identify changes in brain regions associated with flight skills. At the voxel level, GMV was utilized as an indicator to quantify the differences in volume between brain regions. Meanwhile, multiple indicators were applied at the surface level, including cortical curvature, MC-WMS, WM-GM percentage, S-Jacobi, and GC-WMS. These indicators were employed to capture changes in the microstructure of the cerebral cortex. In addition, correlation analyses were conducted to explore the relationship between flight training and behavioral tests. The indicators extracted from each distinct brain region were correlated with scores from the Berg Card-Sorting Test-64 (BCST).

The central aim of this study was to explore the effects of flight training on the brain structure and cognitive function of flying cadets. This study not only offers a novel perspective for understanding the special occupational group characterized by highly specialized skills and strict adherence to disciplines and regulations, but also serves as a vital reference for investigating the changes in brain structure and function of individuals with high physical and psychological demands in high-risk environments. Additionally, this study may contribute to the optimization of the selection and training programs for future flying cadets, thereby facilitating the design of more efficient flight training curricula.

## Materials and methods

### Participants

In this study, 78 students from the Civil Aviation Flight University of China (Guanghan, China) were recruited from June 2022 to October 2022 and divided into the flying cadet group and the general student group. The flying cadet group comprised 39 individuals, all of whom had completed a minimum of 230 hours of flight training, as detailed in Table 1. The control group consisted of general college students who were enrolled concurrently but lacked any experience in flight training. This study established clear inclusion and exclusion criteria. Inclusion criteria included: a) no metallic objects in the body; b) in good health at the time of participation in the experiment; c) all were male; d) aged 21–25 years; e) right-handed; f) all have a bachelor’s degree in engineering. Exclusion Criteria included: a) history of psychiatric disorders; b) have drug or alcohol dependence; c) history of traumatic brain injury, cerebrovascular disease, and chronic pain. This study adhered strictly to the ethical principles outlined in the Declaration of Helsinki and received approval from the Ethical Committee of the University of Electronic Science and Technology of China, under approval number 1420200408–07. All participants were thoroughly briefed about the trial, participated voluntarily, and completed an informed consent form before the commencement of the trial.

**Table 1:** Comparison of demographic data between the two groups.

	Flying cadets (n = 39)	Controls (n = 37)	*T*-value	*P*-value
Age (years)	22.85±0.96	22.38±0.92	2.16	0.49
Gender (% male)	100%	100%		
Education (years) ^a^	16	16		
Handedness (% right)^b^	100%	100%		
Total flight time (hours)	240.56±8.74			

Note: Data were expressed as mean ± standard deviation; ^a^ All the students had obtained a bachelor’s degree in engineering; ^b^ Right-handedness expressed as a percentage of the total participants.

### Data acquisition

All sMRI data in this study were acquired using a 3-T MRI scanner (DISCOVERY MR 750, GE Healthcare, Waukesha, WI, US) at the Magnetic Resonance Imaging Research Center of the University of Electronic Science and Technology of China. T1-weighted images were acquired by three-dimensional (3D) destructive gradient echo pulse sequence with the following parameters: repetition time (TR) = 5.952ms; echo time (TE) = 1.964ms; flip angle = 9°; field of view (FOV) = 256mm×256mm; matrix = 256×256; number of slices = 154; the voxel size = 1mm×1mm×1mm.

The Psychological Experimentation Building Language (PEBL) offers a platform for evaluating psychomotor abilities, encompassing 94 computerized psychological tests [13]. Out of these 94 tests, the BCST was selected to evaluate participants’ cognitive function. The BCST requires participants to sort cards according to rules that involve variations in quantities, color, and the consistency of shapes. The entire test lasted approximately 8 minutes, with the classification rules updating for every 10 consecutive correct answers provided by the participant during the test [6]. The test primarily measures advanced cognitive processing abilities and evaluates participants’ problem-solving abilities.

### MRI pre-processing

**Fig 1:**
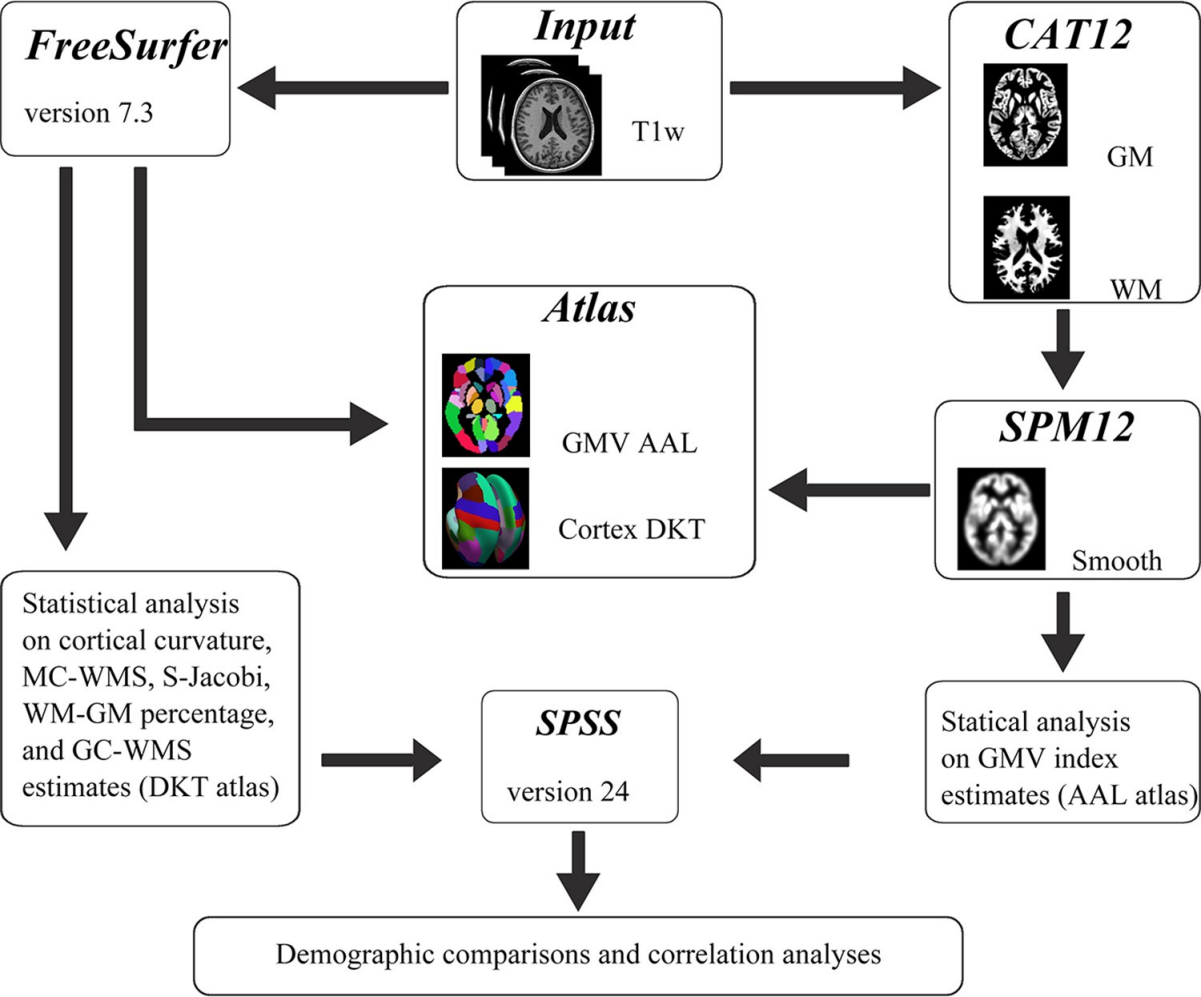
Flow chart of the study. Initially, sMRI imaging was performed with a scanner in the two groups. Subsequently, FreeSurfer software and CTA12 toolkits were used for preprocessing, and the GM images were smoothed using SPM12. Morphological indicators of participants were then obtained. Structural differences between the two groups at the voxel level and at the surface level were compared by two-sample t-test, and the results of the differences were superimposed on a template for presentation. Finally, the relationship between morphological indicators and behavioral tests was assessed.

The flowchart of this paper was depicted in Fig 1. The MRI data were pre-processed and analyzed using the Computational Anatomical Toolbox (CAT12, http://dbm.neuro.uni-jena.de/cat/) and the Statistical Parametric Mapping 12 (SPM12, http://www.fil.ion.ucl.ac.uk/spm/software/spm12) in MATLAB R2013b toolkit. Firstly, T1-weighted images were segmented into white matter (WM), gray matter (GM), and cerebral spinal fluid (CSF). Secondly, spatial normalization was carried out using the Montreal Neurology Institute (MNI) template. Subsequently, the total intracranial volume (TIV) was calculated, and the normalized GM images were subjected to Gaussian smoothing with an 8×8×8mm^3^ kernel, which was utilized for subsequent VBM analysis [14].

FreeSurfer 7.3 software package (http://surfer.nmr.mgh.harvard.edu/) was utilized for cortical segmentation, reconstruction, correction, and registration of T1-weighted images. The recon-all command was employed for automated processing [15], with an average computation time of approximately 8 h per participant. The whole process was briefly conducted as follows [16]: format conversion, motion correction, affine transformation, Talairach transformation, intensity normalization, automated anatomical correction, surface inflation, 10 mm full-width at half maximum smoothing, and the creation of cortical surface indices, such as curvature, MC-WMS, S-Jacobi, which were utilized for the subsequent SBM [17].

### Statistical analysis

In this study, demographics and cognitive function assessment were analyzed using IBM SPSS Statistics v.24 software (https://www.ibm.com/es-es/analytics/spss-statistics-software). For the VBM analysis, the SPM12 statistical analysis toolkit in MATLAB 2013b was employed to compare GM structural differences between the two groups using a two-sample t-test, with age and TIV as covariates. The Gaussian random field (GRF, P < 0.05) method [6] was employed to correct for multiple comparisons, enabling the identification of specific cerebral regions that differed between the flying cadet group and the control group. These findings were subsequently superimposed onto a template for visualization. Additionally, Pearson correlations were analyzed between voxel level indicators of regional differences and behavioral tests.

For SBM analysis, two-sample t-test was performed using the mri_glmfit command in FreeSurfer7.3 to explore differences in cortical surfaces between the two groups. After applying Monte Carlo block level correction (MCBLC, P < 0.05), the differences in brain regions between the flying cadet group and the control group were identified. These differential findings were then visualized by overlaying them onto a template for presentation. At the same time, Pearson correlations were analyzed between cortical surface level indicators of regional differences and behavioral tests.

## Results

### Demographic and clinical characteristics

There were 39 flying cadets and 37 general college students included in this study, none of whom were excluded due to excessive head movement. Demographic parameters were detailed in Table 1. The results indicated that there was no significant difference between the flight cadet group and the control group in terms of age (*T* (74) = 2.162, P = 0.487), gender, education level, and handedness. In behavioral tests, there was no significant difference between the two groups in any of the areas except for accuracy, as shown in Table 2.

**Table 2:** Differences in behavioral tests between the two groups.

	Flying cadets (n = 39)	Controls (n = 37)	*T*-value	*P*-value
BCST_total accuracy (%)	78.97±7.32	71.79±13.47	2.91	0.005
BCST_reaction time (s)	1.90±0.60	1.99±1.46	-0.36	0.72
BCST_perseverative response	18.08±4.86	16.70±5.58	1.15	0.26
BCST_perseverative error	7.03±3.18	6.86±3.07	0.22	0.82

**Notes:** Data were expressed as mean ± standard deviation, * P < 0.05, where BCST is Berg Card-Sorting Test-64.

### Changes in GMV

**Fig 2:**
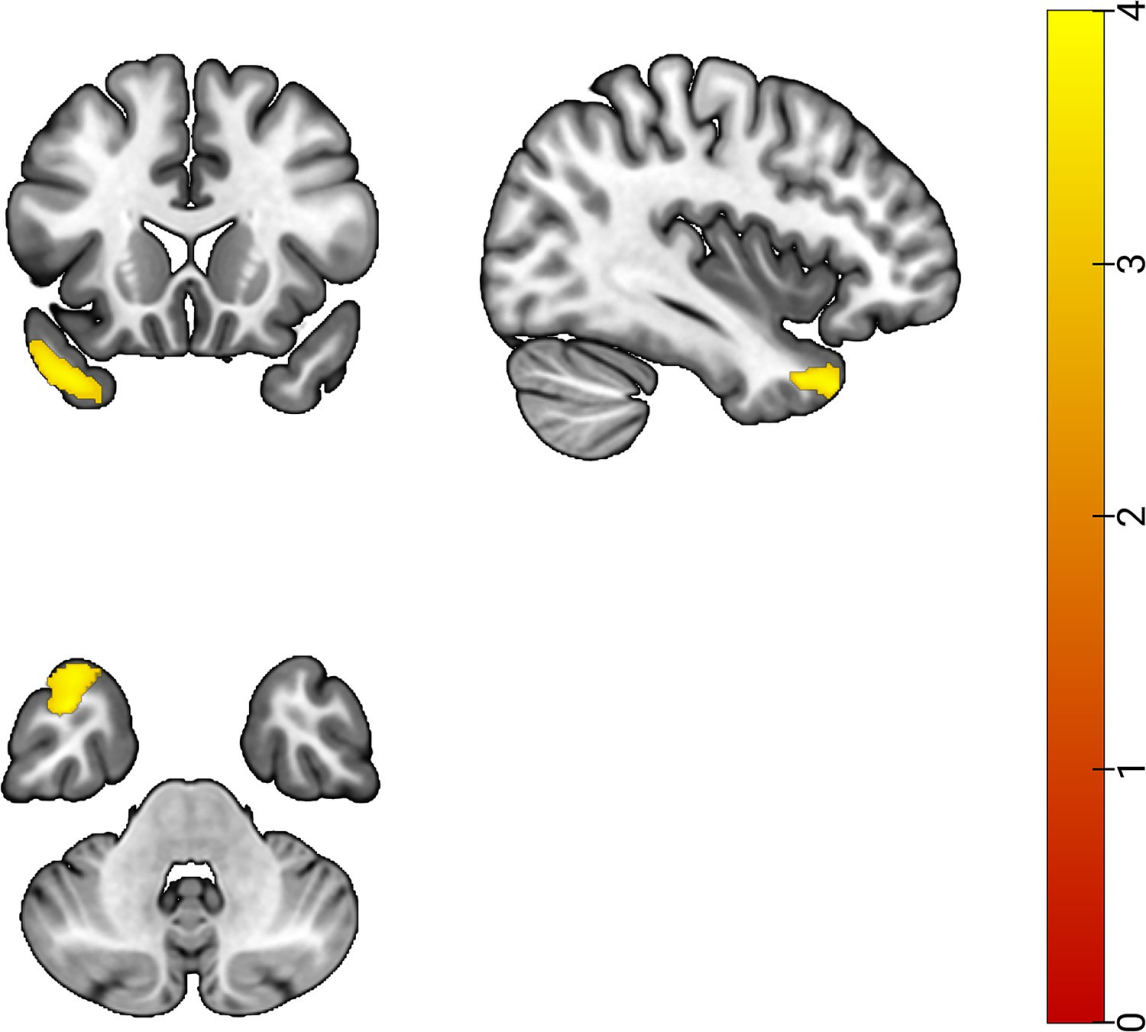
Plot of differences in brain GMV between flying cadets and general college students (GRF corrected, p<0.05, initial threshold p<0.001).

The present study focused on flight cadets and general college students, utilizing VBM for the analysis of T1-weighted data and segmentation into GM, WM, and CSF. Statistical models were constructed using the SPM12 toolkit, and two-sample t-tests were conducted on the two sets of GM data, incorporating age and TIV as covariates. The findings revealed a statistically significant reduction in GMV within the left temporal pole: middle temporal gyrus region among flying cadets compared to the control group, illustrated in Fig 2 and Table 3.

**Table 3:** Differences in regional GMV between the two groups.

Conditions	Cerebral regions (AAL)	Centre (MNI)			Cluster voxels
		X	Y	Z	
Flying cadets < Controls	^a^Temporal_Pole_Mid_L	-39	9	-35	554

**Notes:** ^a^Temporal_Pole_Mid_L denotes the “left temporal pole: middle temporal gyrus region” in the AAL atlas; MNI stands for the Montreal Neurological Institute.

### Cortical morphological changes

**Fig 3:**
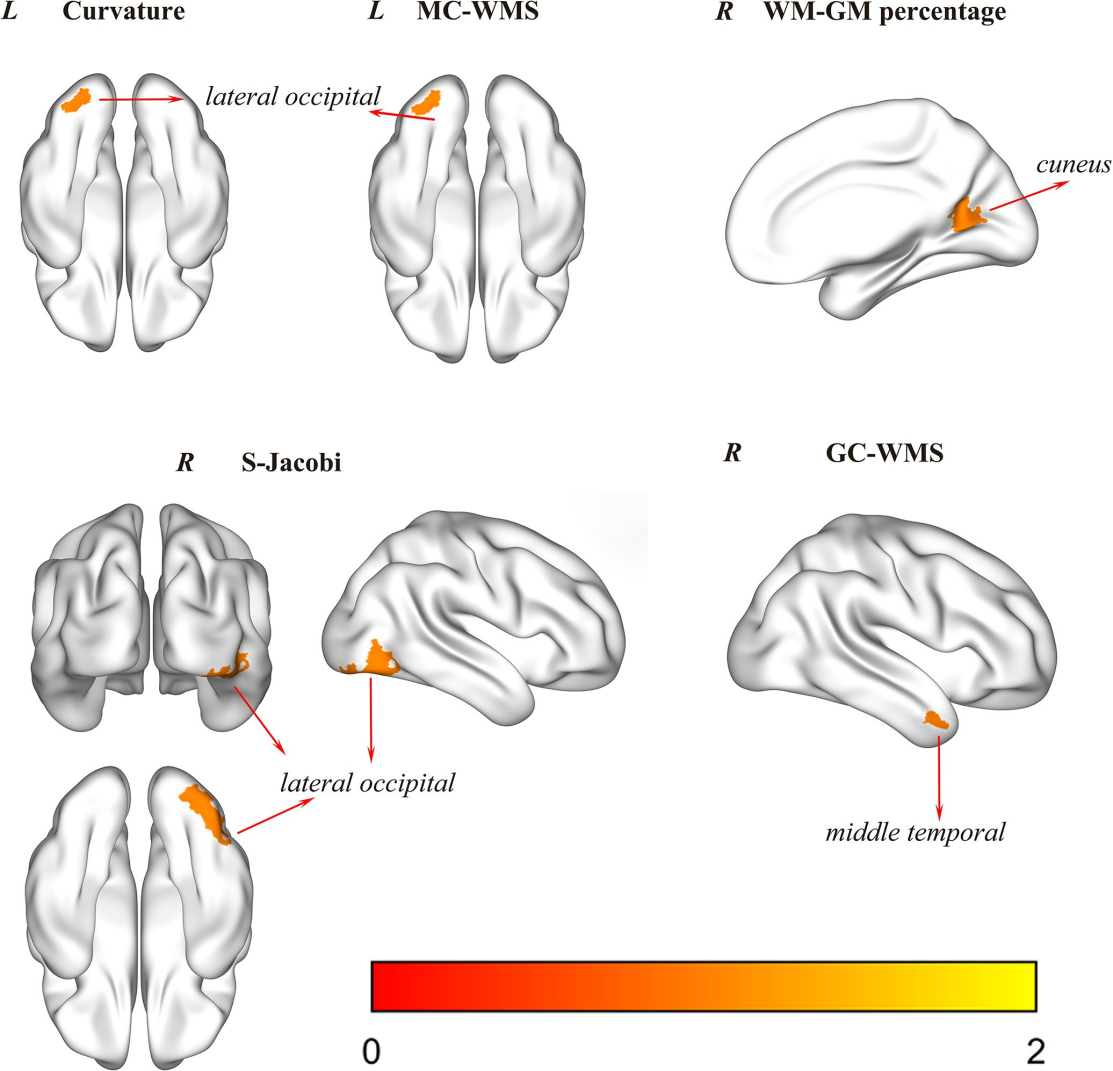
The comparison of differences in cerebral cortex structure between flying cadets and general college students. *L* stands for the left hemisphere, and *R* stands for the right hemisphere. The curvature, MC-WMS, and S-Jacobi in the lateral occipital region were significantly higher compared to the control group (MCBLC, P < 0.05). Furthermore, the WM-GM percentage in the cuneus region and the GC-WMS in the middle temporal region were also significantly higher than in the control group (MCBLC, P < 0.05). MC-WMS stood for mean curvature of the white matter surface, S-Jacobi stood for surface Jacobi, WM-GM percentage represented the proportion of white matter to gray matter on the surface, and GC-WMS denoted the Gaussian curvature of the white matter surface.

**Fig 4:**
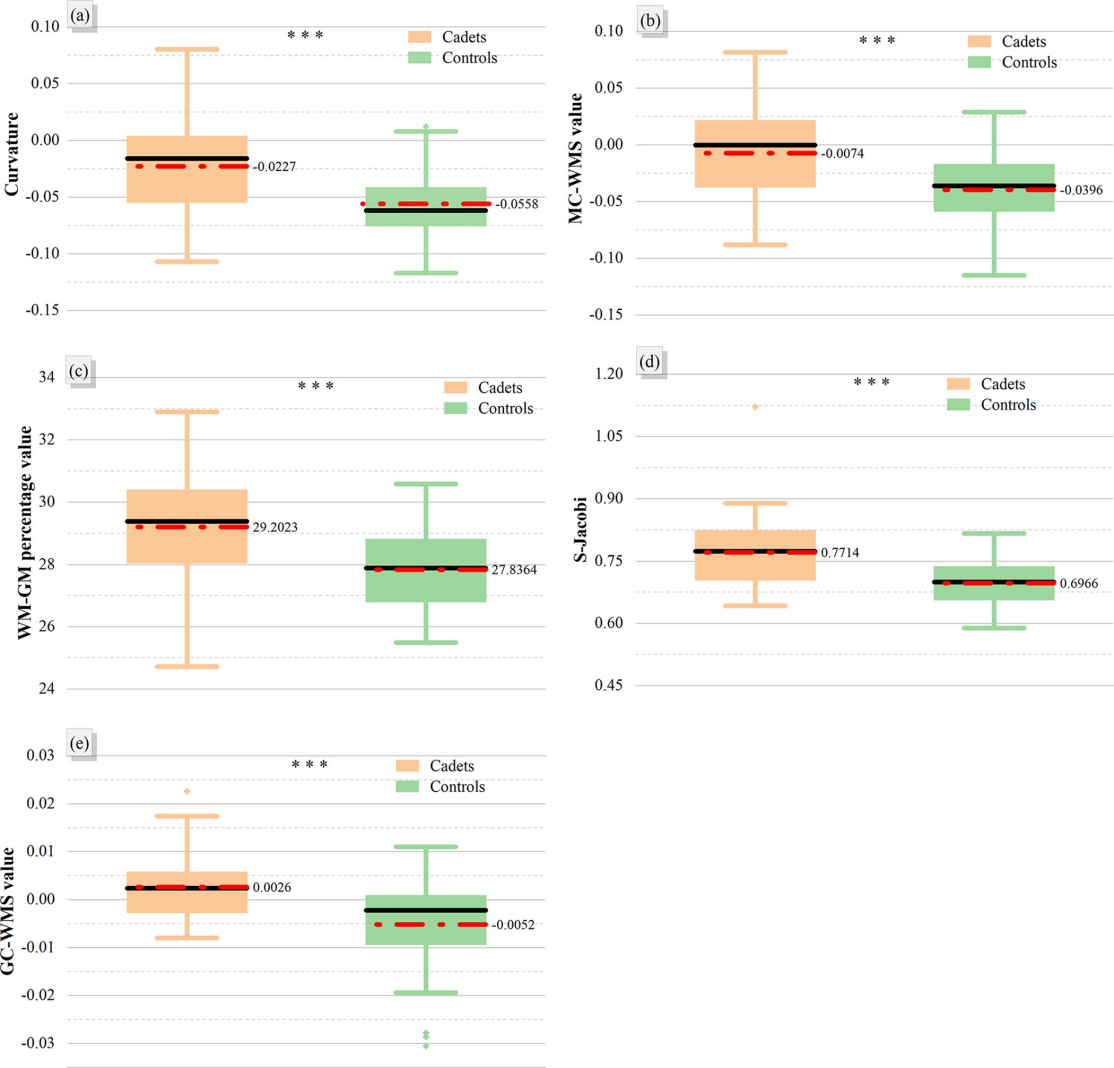
Comparison of cerebral cortical indicators between flying cadets and general college students. (a) curvature information for the lateral occipital region; (b) MC-WMS information for the lateral occipital region; (c) WM-GM percentage information for the cuneus region; (d) S-Jacobi information for the lateral occipital region; (e) GC-WMS information for the middle temporal region. Three stars represented P<0.001, red dotted lines and values represented the mean values, and black lines indicated the median values.

This study employed SBM to investigate cortical morphological changes in participants. Differences between the two groups in curvature, WM-GM percentage, MC-WMS, S-Jacobi, and GC-WMS indicators were compared using age as a covariate, as shown in Table 4, Figs 3 and 4. Additionally, no significant regions of difference were observed in the two widely utilized metrics: cortical thickness and cortical surface area.

**Table 4:** Cortical regions with significant component differences in cortical morphological features.

Measure		Annotation	Max	VtxMax	Peak-MNI			CWP	-log_10_ *P*-value
					x	y	z		
LH	Curvature	Lateral occipital	3.1927	79777	-32.8	-80.1	-11.9	0.0386	1.4
	MC-WMS	Lateral occipital	3.0523	79778	-32.0	-81.0	-12.3	0.0312	1.5
RH	WM-GM percentage	cuneus	3.6567	15206	18.8	-65.3	9.6	0.0001	4
	S-Jacobi	Lateral occipital	3.9732	144001	46.0	-65.2	-10.9	0.0001	4
	GC-WMS	Middle temporal	4.5685	163642	55.4	-1.3	-30.8	0.0247	1.6

**Notes:** L stood for the left hemisphere, and R stood for the right hemisphere. MC-WMS stood for mean curvature of the white matter surface, S-Jacobi stood for surface Jacobi, WM-GM percentage represented the proportion of white matter to gray matter on the surface, and GC-WMS denoted the Gaussian curvature of the white matter surface.

### Correlation analysis

**Fig 5:**
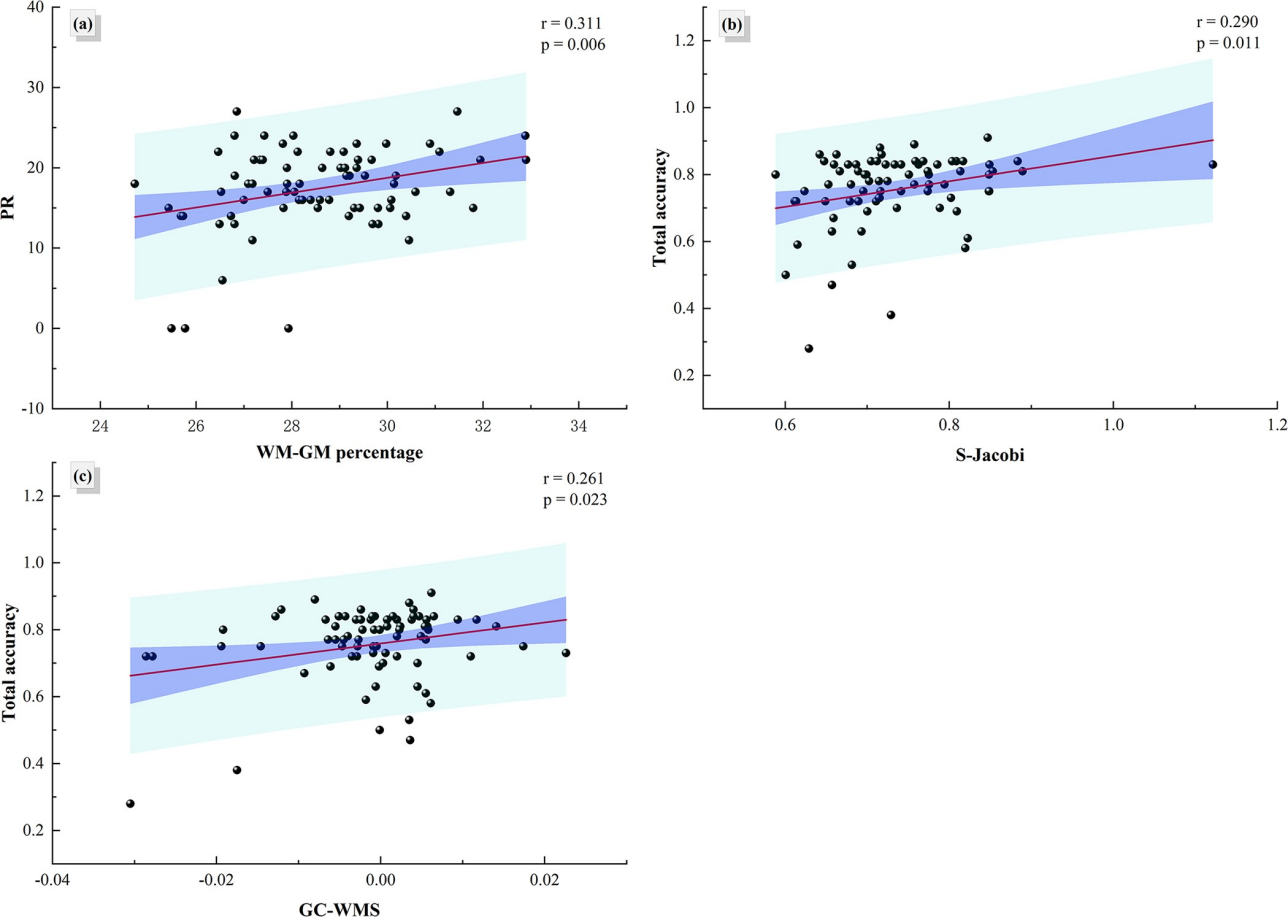
Correlation of extracted indicators with behavioral tests in differentiated cortical regions (p < 0.05). (a) Detailed information for the cuneus region; (b) Detailed information for the lateral occipital region; (a) Detailed information for the middle temporal region.

Correlation analyses using the BCST data, incorporating the extracted indicators in the regions of discrepancy described above, showed no significant correlation between GMV and behavioral tests. However, certain cortical regions exhibited significant correlations between the extracted indicators and behavioral tests. Specifically, the WM-GM percentage of the cuneus region was positively correlated with PR (r = 0.311, P = 0.006). The S-Jacobi value of the lateral occipital region was positively correlated with total accuracy (r = 0.290, P = 0.011). The GC-WMS value of the middle temporal region was positively correlated with total accuracy (r = 0.261, P = 0.023). as displayed in Fig 5.

## Discussion

In this study, the GMV and cortical morphological parameters of the participants’ brains were quantified through VBM and SBM analyses. The results indicated a decrease in GMV at the voxel level in the left temporal pole: middle temporal gyrus region among flying cadets, as illustrated in Fig 2 and detailed in Table 3. Additionally, at the cortical surface level, there was a significant increase in the curvature, MC-WMS, and S-Jacobi in the lateral occipital region of the flying cadets. Furthermore, there was a notable increase in the WM-GM percentage in the cuneus region, as well as an increase in GC-WMS in the middle temporal region, as depicted in Fig 3 and detailed in Table 4. Further analysis demonstrated a correlation between these regional differences and the results of behavioral tests, as illustrated in Fig 5.

Previous studies have indicated that Adamson, M. M. et al. identified a correlation between hippocampal volume and high levels of professional performance [10], whereas Xu, K et al. observed that flight training notably augmented GMV in both the lingual gyrus and fusiform gyrus [12]. Furthermore, Cao, Y. et al. unveiled that the lateralization of cortical structures within the cerebral hemispheres was more pronounced among pilots, as evidenced by cortical thickness analysis [11]. However, the results of the present study differed from these findings, which may stem from two main factors. Firstly, the subjects of this study were flying cadets who had accumulated at least 230 hours of flight time, unlike the experienced pilots in previous studies. Secondly, this study employed a variety of morphological metrics, including GMV, curvature, MC-WMS, WM-GM percentage, S-Jacobi, and GC-WMS, to examine the effects of flight training on brain structure from multiple perspectives. In contrast, early studies have mostly focused on the analysis of single metrics, such as GMV or cortical thickness. In the training of flight cadets, it is important to establish a solid theoretical foundation, enhance awareness of flight safety, and achieve proficiency in flight technology [18]. The main subjects of the training include take-off, climb, cruise, descent, and landing, which require trainees not only to observe the external environment but also to closely monitor various instrument information (such as airspeed, wind direction, etc.) and maintain precise control of the aircraft’s attitude [19]. During training, trainees are required to continuously review and consolidate the skills and experience they have acquired to foster the development of instinctual responses. Simultaneously, it is essential to accurately assess the velocity of the object’s motion and execute the corresponding flight operations as per the provided instructions to enhance the execution of the flight mission.

Through VBM analysis, significant differences in brain structure between flight cadets and general college students were evident, primarily localized in the left temporal pole: middle temporal gyrus region of the temporal lobe, as depicted in Fig 2 and detailed in Table 3. Research has demonstrated that the temporal pole, situated at the anterior end of the temporal lobe, plays a crucial role in semantic memory processes [20]. The middle temporal gyrus, situated deep within the temporal lobe, is closely linked to advanced functions such as language comprehension, memory formation, emotional responses, and social cognition [21]. It also plays a significant role in semantic parsing and lexical comprehension [22]. Additionally, the left temporal pole: middle temporal gyrus region is involved in the processes of memory storage and retrieval [23]. According to existing research, professional training and extensive learning experiences can exert profound influence on brain structure and function [24–26], particularly in certain critical brain regions, potentially leading to alterations in GMV [27]. For flying cadets, significant changes have been observed in their brain GM. This change may arise from the continual repetition and application of the skills and knowledge acquired during flight training, enabling adaptation to varying training content and environments. Flying cadets are tasked with interpreting the meaning of various flight instructions and executing precise flight maneuvers accordingly. Previous studies have shown significant changes in the GMV in the brains of pilots after undergoing flight training [12]. Therefore, it can be presumed that as the GMV in the left temporal pole: middle temporal gyrus region progressively decreases, flying cadets may progressively improve in flight-related skills. With continued flight training and adaptation to the flight environment, it is hypothesized that the GMV of flying cadets will gradually stabilize and eventually correspond to their level of flight ability. This process not only reveals the brain’s adaptive adjustment to the learning of specialized skills but also demonstrates the remarkable plasticity of the human brain.

According to SBM analysis, significant differences in cortical regions were observed between flying cadets and general college students, particularly in the middle temporal, lateral occipital, and cuneus regions, as depicted in Fig 3. Prior research suggests that the middle temporal region can play a pivotal role in directly encoding object speed [28], and it is associated with memory performance[29]. Furthermore, the left middle temporal region is found crucial in processing scores [30], because the cognitive processing of scores requires semantic retrieval to extract mathematical rules [31–33]. Therefore, the left middle temporal region might play a notable role in semantic processing related to mathematics [34–37]. Additionally, the left posterior middle temporal region has been found to be involved in processing action-related meanings, exhibiting a lower response to nouns compared to verbs that are contextually less ambiguous verbs [38]. Studies demonstrate that the right middle temporal region may play a crucial role in face recognition [39].Furthermore, the posterior middle temporal region is associated with comprehension behavior [40], semantic processing [41–43], high-level sound processing [44–46], and syntactic processing [47]. Among flying cadets, significant changes are observed in their middle temporal region, possibly because during flight training they frequently need to assess object velocities and parse the semantics of flight commands. Therefore, it can be hypothesized that the GC-WMS in the middle temporal region of flying cadets may gradually increase as flight training progresses. This change may enhance their ability to assess and calculate the speed of objects, particularly during critical phases such as take-off and landing. Concurrently, it may also facilitate their improvement in areas such as semantic comprehension and syntactic processing.

It is pointed out that the lateral occipital region can be a key region for object perception [48], involving vision [49], touch [50], and hearing [51]. As part of the visual cortex [52], it integrates visual and non-visual information (such as language and reading) and transmits this information to non-visual cerebral parts [53,54]. This region is previously considered as the center for shape processing in human vision [51], highlighting its importance for object recognition [55–57]. Among flying cadets, their lateral occipital region also shows significant changes, likely because during flight training they are required to frequently observe their surroundings and specific landmarks, and to execute complex flight maneuvers. Therefore, it can be hypothesized that the curvature, MC-WMS, and S-Jacobi metrics in the lateral occipital region of flight trainees may gradually increase as their flight training progresses. This change may enhance their perceptual abilities in vision, touch, and hearing, particularly in the domain of object recognition. In addition, the cuneus region located in the occipital lobe of the brain plays a key role in visual selective attention [58] and visual spatial information processing [59,60]. Studies have shown that the cuneus is involved not only in visual processes but also in integrating somatosensory information with other sensory stimuli. Additionally, it is associated with cognitive processes such as attention, learning, and memory [61]. Meanwhile, the precuneus, as part of the multisensory vestibular cortex, plays a pivotal role in processing vestibular information, which is essential for spatial orientation and spatial perception [62–65]. In flying cadets, significant changes have been observed in the cuneus region, likely due to their requirement to allocate attention based on the importance of information displayed on the panel during flight training. Additionally, this region is involved in spatial orientation and spatial perception during flight. Therefore, it can be inferred that the WM-GM percentage in the cuneus region of flying cadets may gradually increase as flight training progresses. These changes may enhance their visual and spatial processing capabilities, particularly in terms of attention allocation.

In the present study, the BCST in PEBL was employed to assess the cognitive abilities of the participants, with a specific focus on evaluating problem-solving abilities. The BCST’s total accuracy, total reaction time, perseverative responses (PR), and perseverative errors (PE) evaluate the problem-solving abilities of flying cadets [66]. PR denotes perseverative responses to old or new rules, without considering feedback; PE is the wrong PR [6]. The overall accuracy of the flying cadets was significantly higher than that of the control group, as shown in Table 2, indicating that the flying cadets could effectively adapt to the rule changes in the test and make accurate judgments. This may be attributed to the heightened sensitivity of flying cadets to alterations in regulations and their improved problem-solving abilities following professional training, resulting in substantial differences in accuracy during the behavioral assessment. In the correlation analysis, the mean WM-GM percentage in the cuneus region is markedly higher in the flight cadet group compared to the control group, and demonstrates a positive correlation with PR, as illustrated in Figs 4 and 5. Therefore, it can be inferred that the increase in WM-GM percentage in the cuneus region of the flying cadets likely strengthens their response to the rule change, thereby enhancing their problem-solving abilities. The correlation analysis also indicates that the mean S-Jacobi value in the lateral occipital region and the mean GC-WMS value in the middle temporal region are significantly higher in the flying cadet group compared to the control group. Furthermore, these values show a positive correlation with total accuracy, as illustrated in Figs 4 and 5. This suggests that the S-Jacobi in the lateral occipital region and GC-WMS in the middle temporal region are elevated in flying cadets, potentially enhancing their problem-solving accuracy. In conclusion, it is speculated that flight training may elicit adaptations in certain brain regions of flying cadets, enabling them to better cope with evolving training content and environments, thus enhancing both their problem-solving abilities and flight proficiency.

There were several limitations to this study. First, this study only collected cross-sectional data and failed to track longitudinal changes in the brain structure of flying cadets. Second, this study did not investigate the influence of various training subjects on the brain structure, particularly their impact on specific brain regions. To address these limitations, future research should involve collecting and analyzing sMRI data from specific flight training trainees both before and after training to enable longitudinal analysis. Additionally, it should explore the effects of different training subjects on brain structure and function. This will contribute to a deeper understanding of how flight training impacts the brain structure and cognitive functions of flying cadets, thereby advancing our knowledge of brain plasticity.

## Conclusions

This study utilized sMRI data analysis at both the voxel and surface levels, employing multiple indicators to investigate the effects of flight training on flying cadets. The findings suggested that flight training could potentially lead to changes in specific brain regions such as the left temporal pole: middle temporal gyrus region, lateral occipital, cuneus, and middle temporal regions. These changes were associated with enhancements in cognitive functions including memory storage and retrieval, semantic retrieval, object speed assessment, spatial orientation, spatial perception, attention allocation, as well as vision and auditory perception. These cognitive improvements are likely to contribute positively to problem-solving and flight abilities among the cadets undergoing such training. In the future, the focused training of specific cerebral regions will significantly influence pilot training. This study provides valuable insights into elucidating the intricacies of brain structure, function, and plasticity, while also facilitating exploration into the neural mechanisms specific to the pilot’s brain.
